# Quality of anti-malarial drugs provided by public and private healthcare providers in south-east Nigeria

**DOI:** 10.1186/1475-2875-8-22

**Published:** 2009-02-10

**Authors:** Obinna Onwujekwe, Harparkash Kaur, Nkem Dike, Elvis Shu, Benjamin Uzochukwu, Kara Hanson, Viola Okoye, Paul Okonkwo

**Affiliations:** 1 Department of Health Administration and Management, College of Medicine, University of Nigeria, Enugu, Nigeria; 2 Health Policy Research Group, Department of Pharmacology and Therapeutics, College of Medicine, University of Nigeria, Enugu, Nigeria; 3 London School of Hygiene and Tropical Medicine, London, UK; 4 Roberta Buffett Center for International & Comparative Studies, Northwestern University, Chicago, USA; 5 Department of Community Medicine, College of Medicine, University of Nigeria, Enugu, Nigeria; 6 University of Nigeria Teaching Hospital, Ituku-Ozala, Nigeria

## Abstract

**Background:**

There is little existing knowledge about actual quality of drugs provided by different providers in Nigeria and in many sub-Saharan African countries. Such information is important for improving malaria treatment that will help in the development and implementation of actions designed to improve the quality of treatment. The objective of the study was to determine the quality of drugs used for the treatment of malaria in a broad spectrum of public and private healthcare providers.

**Methods:**

The study was undertaken in six towns (three urban and three rural) in Anambra state, south-east Nigeria. Anti-malarials (225 samples), which included artesunate, dihydroartemisinin, sulphadoxine-pyrimethamine (SP), quinine, and chloroquine, were either purchased or collected from randomly selected providers. The quality of these drugs was assessed by laboratory analysis of the dissolution profile using published pharmacopoeial monograms and measuring the amount of active ingredient using high performance liquid chromatography (HPLC).

**Findings:**

It was found that 60 (37%) of the anti-malarials tested did not meet the United States Pharmacopoeia (USP) specifications for the amount of active ingredients, with the suspect drugs either lacking the active ingredients or containing suboptimal quantities of the active ingredients. Quinine (46%) and SP formulations (39%) were among drugs that did not satisfy the tolerance limits published in USP monograms. A total of 78% of the suspect drugs were from private facilities, mostly low-level providers, such as patent medicine dealers (vendors).

**Conclusion:**

This study found that there was a high prevalence of poor quality drugs. The findings provide areas for public intervention to improve the quality of malaria treatment services. There should be enforced checks and regulation of drug supply management as well as stiffer penalties for people stocking substandard and counterfeit drugs.

## Background

People seek treatment for malaria from public sector facilities and a range of formal and informal private sector facilities [1,2]. Around 60% of all malaria episodes in sub-Saharan Africa (SSA) are initially treated by private providers, mainly through the purchase of drugs from shops and drug peddlers [1]. The "informal private sector", such as patent medicine dealers, is a main source of anti-malarial drugs [3,4], but the quality of treatment that they provide is suspect [1]. However, these treatments are often inconsistent with national treatment guidelines: they may include counterfeit drugs, drugs of poor quality, as well as incorrect dosing and irrational prescription practices [4]. A counterfeit formulation is one that is "deliberately and fraudulently mislabelled with respect to identity and/or source. Counterfeiting can apply to both branded and generic products and counterfeits may include products with the correct ingredients or with the wrong ingredients, without active ingredients, with insufficient active ingredient or with fake packaging" [5]

Drug quality in public and private outlets may be problematic. A previous study in Nigeria assessed the quality of drugs from retail outlets and pharmacies, and attributed problems to a lack of quality control in manufacture and degradation during storage [6].

A major problem with the treatment of malaria is the high level of treatment failures resulting in the large part from the high prevalence of counterfeit drugs bought by the patients [7-9]. Anti-malarials, are among the most widely consumed drugs in tropical countries that have been particularly targeted by counterfeiters and of the 12 anti-malarial drugs used in the world today, eight have been counterfeited [7]. Published estimates of the global prevalence of counterfeit drugs range from 1% to 50% and there is evidence of 206 cases of counterfeit anti-infectives from 38 countries [8]. The widespread prevalence of counterfeit anti-malarials is of great public health concern [7,8]. Also, lack of knowledge of counterfeits and appropriate preventive measures, together with poor dissemination of information among health workers and the public, make their detection difficult [8].

Although the official treatment policy has been changed as per WHO recommendations to the use of artemisinin-based combination therapy (ACT) as first-line treatment for malaria in Nigeria, the reality on the ground is the continued production, deployment and use of monotherapies, such as chloroquine (CQ), sulphadoxine-pyrimethamine (SP), quinine (QU), artesunate and dihydroartemisinin, in both public and private facilities, especially by patent medicine dealers (vendors) in Nigeria and other African countries. Artemisinin monotherapy remains common in Africa [9]. Researchers have evaluated the quality of CQ, quinine, SP, amodiaquine and proguanil formulations sold in the market in various parts of Africa, including eastern part of Congo DR and Kenya [10-12]. Good quality anti-malarial drugs are often misused in treating malaria because of under-dosing and poor adherence, which could lead to treatment failures and development of drug resistance. The use of counterfeit or substandard monotherapies further endangers malaria chemotherapy.

There is paucity of information about the quality of anti-malarials in many sub-Saharan African (SSA) countries, such as Nigeria. Most of the evidence about quality of anti-malarials has come from South-East Asia. However, in a six-country study that highlighted the availability and relative quality of anti-malarials in Africa's private sector, found that over 35% (73/210) of tested samples were substandard [9]. In Nigeria, 36% of sampled anti-infectives contained quantities of active ingredients outside pharmacopoeial limits [6,8]. Also, from a random sample of 5% (n = 581) of Nigerian pharmacies, 48% of anti-infectives contained active ingredients outside pharmacopoieal limits [6,8]. In some cases, the drugs may contain more of the stated active ingredients, which could lead to adverse events as was found in Nigeria where 94 of 160 (59%) of anti-malarials tested contained 110% or more of the stated active ingredients [8,13].

There is little existing knowledge about actual quality of drugs provided by different providers in Nigeria and in many SSA countries. A search of the medical literature yielded only 43 primary published research reports concerning counterfeit drugs in the world [8]. Some researchers found 21 peer-reviewed articles and three reports on the quality of anti-malarial drugs in Africa [14]. Failing products more often originated or were claimed to originate from poorer parts of the world with weaker regulatory systems [9]. The critical finding by some researchers was that most anti-malarial drugs pass the basic tests for pharmaceutical dosage forms such as the uniformity of weight for tablets and the content test, but that *in vitro *product dissolution is the main problem [12,14].

Over the past decade, the massive public health problem of counterfeit and substandard drugs has become more manifest, leading to serious clinical consequences to patients, such as increased morbidity, mortality, and drug resistance, which leads to spurious reporting of resistance and toxicity and loss of confidence in healthcare systems [8,15,16]. Other studies looking at a broader range of diseases in Nigeria found widespread inappropriate drug use, low quality of treatment, and ineffective regulation [17-21].

The information generated by this study will help design policy measures to strengthen the treatment component of the malaria control strategy. Such information is especially important for improving malaria treatment, especially in light of the change of first-line drug to ACT in the country. The information will also lead to the development and implementation of targeted actions designed to remedy problems found with respect to quality of treatment. These remedial actions could include improved training of public and private sector providers, strengthened regulation, and consumer education.

## Methods

### Study area

Anambra State, Southeast Nigeria was chosen for the study. South-east Nigeria is one of the most important sources of anti-malarial drugs in Africa and the "bridge-head" market in Onitsha, Anambra State is at the centre of this trade. The state has a high malaria transmission rate all year and the annual incidence rate is 10 to 35%. Six sites were chosen for the study. These were the three largest urban centres (Awka (state capital), Nnewi and Onitsha), from each of the three senatorial zones and one rural local government area (LGA), randomly selected from each senatorial zone (Njikoka, Aguata and Ogbaru). Then, one community from each of the three rural LGAs: Enugwu-Ukwu (Njikoka LGA), Ekwulobia (Aguata LGA) and Okpoko (Ogbaru LGA) was selected using two-stage sampling, by first stratifying the communities according to whether they have a general hospital and then randomly selecting the sites from those that have general hospitals. Each site area has a full complement of providers from hospitals to itinerant drug providers and herbalists.

### Sampling and sample size

The sample size was determined by considerations of the range of providers and feasibility and was selected from a broader study of nature of malaria treatment provision. In the broader study, 50 providers (public and private) in each urban and 25 in each rural area were selected, which gave a total of 225 providers. The quality of drugs was assessed through the purchase of anti-malarial drugs from a random sample of 20% of the 225 selected providers. They were spread out across the different levels of providers, but all existing public providers in each study area were included in the study, because there were not many of them. The sampling frame was providers using orthodox drugs to treat patients and they included all levels of care in public facilities and private providers. Orthodox drugs refer drugs derived from biomedical science and include tablets, syrups, suspensions and injections (in contrast to herbs and homeopathic drugs). The providers were divided into low-level providers and high/medium level providers. The low-level providers included patent medicine dealers, mixed goods shops and maternity homes. The medium/high level providers included public hospitals, private hospitals, pharmacy shops and primary healthcare centres. Pharmacy shops are legal drug outlets with an in-house pharmacist, whilst patent medicine dealers are also legal drug outlets without an in-house pharmacist.

### Collection/purchase of drugs

The drugs were purchased from both low level and high/medium level providers. The major drugs that are used in the co-packaging of ACT, as well as the most common anti-malarial were purchased. All drugs were purchased in tablet form and the number of each tablet purchased was determined by the type of anti-malarial and the recommended single dose for treatment of malaria. The drugs were artesunate, dihydroartesinin, SP, QU and CQ tablets. The drugs were purchased in the following quantities: artesunate – 12 tablets, dihydroartesinin – eight tablets, SP – three tablets, quinine – 10 tablets, and CQ – 10 tablets. Researchers posed as clients to buy the drugs from pharmacy shops and patent medicine dealers (PMDs) or vendors provided such drugs that had shelf lives of more than two years. However, in the case of hospitals and a few pharmacy shops, the researchers explained the purpose of the study and requested that the providers sell the anti-malarials to them, which all of them did. In some cases, the hospitals donated the samples. Drugs of different lot and batch numbers were bought/collected and the samples for testing were randomly selected from those. The drugs that were in packets were left in their packets, but those that were bought in packs were safely stored in medicine containers at room temperature (28°C to 32°C). Hence, efforts were made to ensure that the drugs were not degraded by inadequate storage conditions, which would have confounded the findings. The drugs were stored for 4.5 months before the analysis. None of the analysed drugs had expired.

### Drug quality by dissolution analyses

The quality of the formulations of SP, quinine and CQ was determined using the *in vitro *dissolution testing protocols using the detailed monograms outlined in the United States Pharmacopeia (USP) and measuring the amount of active component using high performance liquid chromatographic (HPLC) analysis [22]. HPLC analysis was carried out at the London School of Hygiene and Tropical Medicine. The test for content assesses the amount of active ingredient measured in a formulation, expressed as a percentage of the label claim; the test for dissolution determines the amount of active ingredient that this released and available for absorption [11]. Poor manufacturing practices, poor storage of a product as well as the use of incorrect excipients will lead to poor dissolution profiles and thus result in compromised bioavailability. Dissolution testing for pharmaceutical products in tablet and capsule form is required by the US Food and Drug Administration (FDA) and increasingly used outside the USA to report on the quality of drug.

#### Dissolution analyses

Tablet dissolution was performed in the Pharma Test PT 017 dissolution apparatus using 6 tablets of each product. Dissolution of all antifolate antimalarial products was carried out using 1 litre of 0.01 M pH 6.8 phosphate buffer solution (sodium hydroxide and potassium dihydrogen orthophosphate, Fisher Scientific) and heated to a temperature of 37°C, with a rotor speed of 75 rpm. Dissolution was carried out for 40 minutes and 500 μl samples were taken at ten-minute intervals during this time. Of this 500 μl sample 200 μl was transferred into a high performance liquid chromatography (HPLC) reaction vial and diluted 1:1 with 200 μl 0.05 M pH 6.8 phosphate buffer solution and transferred into the HPLC machine for analysis. Dissolution of CQ was performed in 900 mls of purified water and heated to a temperature of 37°C with a rotor speed of 100 rpm for 60 minutes. At ten-minute intervals, 500 μl samples were taken and from each of these 200 μl was transferred for HPLC analysis after a 1:1 dilution with purified water. The tablets were subjected to dissolution in 900 mls of 0.1 Molar HCl and heated to a temperature of 37°C, with a rotor speed of 100 rpm. Dissolution was carried out for 1 hour with 500 μl samples taken at ten-minute intervals during this time. From each 500 μl sample 200 μl was transferred into a HPLC reaction vial and diluted 1:1 with 200 μl 0.5 M HCl and subsequently transferred into the HPLC machine for analysis.

### Quantity of active ingredient

**Figure 1:**
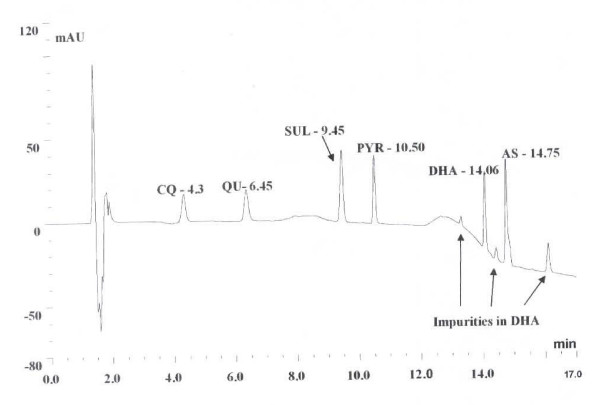
**HPLC chromatogram showing the separation of mixture of standards of chloroquine (CQ), quinine (QU), sulphadoxine (SUL) and pyrimethamine (PYR) all at 10 μg/ml; dihydroartemisinin (DHA) and artesunate (AS) at 4 mg/ml**.

Drug quality was assessed by comparing the amount of active ingredient in the eluents of each dissolution sample against a known concentration of the standard for CQ, QU, SUL and PYR after HPLC analysis (see chromatogram in Figure 1 for the separation for each compound). Information about the source, packaging or appearance of each product was not known by the investigators prior to analyses of the tablets for quality.

After the dissolution and HPLC analyses the samples were classified as good quality or substandard based on the amount of active ingredient detected listed in Table 1, below. Similarly in the absence of an official monograph for the dissolution profile of artemisinin derivatives – artesunate (AS) and dihydroartemisinin (DHA) – concentrations were measured directly by crushing the tablets and adding 20 ml of methanol to obtain a 2.5 mg/ml solution of AS or DHA per tablet. This solution was then analysed on HPLC to confirm the amount measured. The amount of active ingredient detected was determined from a calibration curve plotted using reference standards of each of the artemisinin derivatives (0–10 mgs/ml)

**Table 1:** Classification for content analysis by HPLC for antimalarial drugs

**Drug**	**Good Quality**
Chloroquine	> 0.208 mg/ml at 45 minutes (if CQ = 250 mg dose)
Quinine	> 0.25 mg/ml at 45 minutes (if QU = 300 mg or 0.08 mg/ml if it is 100 mg)
Sulfadoxine	> 0.3 mg/ml at 30 minutes
Pyrimethamine	> 0.015 mg/ml at 30 minutes
ARTs	> 95% of stated concentration

For SP, USP stipulates that 60% of each component must be detected in the dissolution media (phosphate buffer 0.05 M; pH 6.8) at 30 minutes (37°C). For quinine an amount of more than 75% must be detected in the dissolution media (0.1 N hydrochloric acid) at 45 minutes (37°C), while for CQ, 75% of the active ingredient should be detected in the dissolution media (water) at 45 minutes (37°C). Dissolution tests and percentage of active ingredients of the drug samples were the major points for data analysis. The final results (Table 1) were expressed as whether or not the drugs failed to meet the tolerance limits stated in the monograms of USP and the proportion of active ingredients that each sample contained. As reported by Bate *et al*, forensic examination of trademarks or product designs were carried out to differentiate between products that were merely substandard and those which were deliberately counterfeited [8].

## Results

### General characteristics of the providers

The basic training and service information of low level healthcare providers and medium/high level providers showed that amongst the low level providers, those in maternity homes had the highest average number of years of formal training for the work that they do. Similarly, it was providers in 'other healthcare facilities' that had the highest number of years of training for the work that they do, whilst providers in private hospitals, the general hospitals and 'others' had the highest level of education for medium/high level providers. All the medium/high level providers were licensed for the work that they do, with the exception of one provider from a Primary Healthcare (PHC) centre. Conversely, 121 (94.5%) of the low level providers were licensed, however the level of education for most patent medicine dealers, specifically for the medicine business, was very low. Overall, there were 128 low-level providers and 97 medium/high level providers.

### Drugs stocked and used by providers to treat malaria

In the survey interview conducted with all 225 selected providers, artesunate monotherapy, chloroquine tablets, chloroquine injection, antibiotics, SP, quinine, ACT and other drugs were stated as the drugs that 30.9%, 70%, 54.6%, 24.7%, 56.7%, 23.7%, 11.3%, 38.1% medium/high level providers most commonly used to treat malaria. The use of artesunate monotherapy was mostly in public (81.8%) and private hospitals (100%) and pharmacies (63.6%) respectively. Also, CQ tablets (90.6%) and SP formulations (84.4%) were the most common drugs used by low-level providers to treat malaria. Only 24.2% and 0.8% of low-level providers used artesunate monotherapy and ACT, respectively to treat malaria. The few instances of known use of ACT by medium/high level providers were in general hospitals and pharmacy shops.

At the time of the interview the medium/high level providers had in stock artesunate monotherapy (29.9%), CQ tablets (61.9%), CQ injection (57.7%), antibiotics (20.6%), SP (45.4%), quinine (22.1%) and ACTs (14.4%). Most of the formulations for artesunate monotherapy and ACT were stocked by the public hospitals and pharmacy shops. Conversely, at the time of interview, just 27 (21.1%) and two (1.6%) of low level providers had in stock artesunate monotherapy and ACT and 88.3% of them stocked both CQ and SP tablets at that time.

### Quality of different anti-malarials in the study area

Table 2 shows that 60 (37%) out of the 225 anti-malarials did not meet the tolerance limits set by USP for the amount of active ingredient when tested for the quality of the formulations. The drugs that did not meet the tolerance limits set by USP either did not contain the active ingredients or contained suboptimal quantities of the stated active ingredient/s. Most of the drugs that did not meet the tolerance limits set by USP were found with respect to quinine (46%) and SP preparations (39%). Table 3 shows the sources of the drugs that did not meet the tolerance limits set by USP by type of provider. A total of 78% of the drugs that did not meet the tolerance limits set by USP were found in private facilities, mostly in patent medicine stores. Similarly, 60% of the drugs that did not meet the tolerance limits set by USP were found in low-level providers, mostly in patent medicine stores. Seven of the quinine tablets contained only chloroquine. One sample of the total of four samples of dihydroartemisinin collected, was a counterfeit and one tablet of CQ contained half the amount of active ingredient, with one CQ sample actually containing nothing which by definition implies it is a counterfeit. Chi-square analysis shows that the difference between the urban and rural areas in drugs that did not meet the tolerance limits set by USP was statistically significant (Chi-square = 51.24, df = 12, p < .0001). Table 4 shows that the quality of drugs, especially SP and quinine were quite bad in the rural areas, when compared with the situation in the urban areas.

**Table 2:** Showing the quality of different anti-malarials in different study communities

	Dihydroartemesinin N = 4 n (%)	Quinine N = 28 n (%)	Sulphadoxine-Pyrimethamine (SP) N = 113 n (%)	Chloroquine N = 56 n (%)	Artesunate N = 24 n (%)	Total N = 225 n (%)
Pass*	3 (75)	15 (54)	69 (61)	54 (96)	24 (100)	165 (73)
Fail*	1 (25)	13 (46)	44 (39)	2 (4)	0 (0)	60 (37)
Total	4 (100)	28 (100)	113(100)	56 (100)	24 (100)	225 (100)
						
Failures						
Public	0 (0)	2 (15)	9 (21)	2 (100)	0 (0)	13 (22)
Private	1 (100)	11 (85)	35 (79)	0 (0)	0 (0)	47 (78)
Total	1 (100)	13 (100)	44 (100)	2 (100)	0 (0)	60 (100)
						
Failures						
Low level	0 (0)	10 (77)	25 (57)	1 (50)	0 (0)	36 (60)
High/med	1 (100)	3 (23)	19 (43)	1 (50)	0 (0)	24 (40)
Total	1 (100)	13 (100)	44 (100)	2 (100)	0 (0)	60 (100)

Note: A failure in the context of the study implies that the tablets did not meet the USP tolerance limits for quality test for active ingredient released during the dissolution test.

**Table 3:** Showing the sources of drugs that failed quality tests by type of provider

	Dihydroartemesinin N = 1 n (%)	Quinine N = 13 n (%)	(SP) N = 44 n (%)	Chloroquine N = 2 n (%)
Patent medicine dealers	0	7 (53.9%)	23 (52.5%)	0
Pharmacy shops	0	3 (23.1%)	13 (29.6%)	0
Private hospital	1 (100)	1 (7.7%)	0 (0)	0
Public Hospital	0	0 (0)	4 (9.1%)	2 (100%)
Primary healthcare centers	0	2 (15.4%)	4 (9.1%)	0
Total	1 (100)	13 (100)	44 (100)	2 (100%)

Χ^2 ^for urban rural differences in failed drugs = 51.24, df = 12, p < .0001.

**Table 4:** Rural urban differences in drug quality

	SP		Quinine		CQ		DHA		AS	
	total	Failed*	total	Failed*	total	Failed*	total	Failed*	total	Failed*

Urban areas	54	20 (37.0%)	16	7 (43.8%)	26	1 (3.9%)	4	1 (25%)	12	0 (0%)

Rural areas	59	26 (44.1%)	12	8 (66.7%)	30	1 (3.3%)	0	0 (0%)	12	0 (0%)

Grand total	113	46 (40.7%)	28	15 (53.6%)	56	2 (3.6%)	4	1 (25%)	24	0 (0%)

Note: Pass or fail implies that the tablets met or did not meet the USP tolerance limits for quality test

## Discussion

There was a high prevalence of many failing anti-malarial drugs in the study area, which is at the heart of pharmaceutical trade in West Africa. Given that the drugs tested where the drugs also mentioned by the providers that were interviewed as the most commonly used drugs for malaria treatment, it leads to many cases of apparent malaria treatment failures, and misdiagnosis of such to be enteric fever as is common in Nigeria. All these lead to economic loss and death as well as increasing cases of drug resistance. It was alarming that many of the drugs that did not meet the tolerance limits set by USP were from licensed providers and that the current prevalence rate is similar (36%) as reported in an earlier study [6]. The results are also similar to the findings in the eastern region of Congo DR, where high prevalence of substandard CQ, QU and SP were found [10], and similarly in Kenya, where there was high prevalence of substandard SP and amodiaquine [11]. In Cameroon, which shares a border with Nigeria, it was found that of the tested CQ (38%), QU (74%) and antifolates (12%) had either no active ingredient, an insufficient active ingredient, the wrong ingredient, or unknown ingredient(s) [23]. These results are similar to those reported from the study from SE Asia, where an increasing high proportion of anti-malarial drugs bought in pharmacies and shops are counterfeit [24]. Poor quality artemisinin monotherapies such as dihydroartemisinin have also been reported from formulations tested in Kenya [14].

Unexpectantly cheap drugs such as SP formulations and quinine, and to a lesser extent CQ, which are most commonly used by the very poor, were counterfeit. Similarly, it was found that 33% of CQ batches were under dosed in eastern region of Congo DR [10]. Hence, as argued by some researchers, the previous suggestions that relatively inexpensive drugs, such as CQ are not commonly faked is not borne out by data [8]. The results compare well with the those from another study where it was found that various substandard therapies and clinically inappropriate mono-therapies remain widely used, with between a quarter half of the products failing basic quality tests [9]. Specifically 33% of artemesinin monotherapies failed thin-layer chromatography tests [9]. The case of high failure rate of quinine is of grave concern because physicians in Nigeria usually rely on this drug for cases of suspected treatment failures to other drugs and in emergencies. Also, the high level of SP formulations that did not meet the tolerance limits set by USP portends danger for pregnant women, as this drug is the mainstay of intermittent-preventive treatment of malaria in pregnancy (IPTp).

The motive for such sustained high prevalence could be the inordinate objective of high profit making by unscrupulous businessmen aided by pharmaceutical manufacturers. It has been estimated that the counterfeit medicine market is worth some US$35–44 billion per year [8]. However, it was stated that numerous factors encourage the counterfeiting of drugs apart from criminal greed [8]. The relatively high cost of genuine medicines, together with their desirability and low availability, give the counterfeiters an economic incentive, facilitated by lack of legislation and enforcement and light penalties [8].

The pattern of low quality of drugs that was found in this study has equity implications for appropriate treatment of malaria in Anambra state and even Nigeria. This is because the private and low-level providers, where the drugs that did not meet the tolerance limits set by USP were available are usually predominantly used more by the poorer/poorest socio-economic status (SES) groups [1]. In Tanzania, there was availability of low quality SP and amodiaquine tablets in wholesale pharmacies [15]. Similarly in Cambodia, it was found that out of 133 drug vendors, 71% and 60% had counterfeit artesunate and mefloquine respectively [8,25]. The low level providers with the drugs that did not meet the tolerance limits set by USP are also usually the places where quality of malaria treatment is usually the lowest [1]. These findings mean that the poorer and most poor SES received the lowest quality of treatment from all ramifications. As such, urgent interventions should be developed and implemented to remedy this inequity, as counterfeit drugs particularly affect the most disadvantaged people in resource poor countries [8].

Improved drug quality for treatment of malaria will require concerted educational intervention for providers and consumers to enhance procurement of good quality drugs and, improved regulation of the drugs. This is especially pertinent because there was greater prevalence of poor quality drugs in the rural areas where there is lack of knowledge and ignorance by both providers and consumers about quality of healthcare is rife, and they also lack awareness of the consequences of counterfeit drugs. In Laos, it was found that 63% of drug sellers and 80–96% of consumers were not aware of the existence of poor quality drugs [8,26]. The finding that some drugs contained the wrong stated active ingredient or contained nothing is quite worrying in the study area used for this study, where *Plasmodium falciparum *causes more than 90% of the malaria. This can lead to increased morbidity and mortality if counterfeit drugs are used for treatment. ACT is expensive and the current policy to use such combinations as first-line drugs implies an urgent need to decrease the spread of counterfeit drugs in the system, otherwise people will be wasting sums of money buying counterfeit ACT. The incidence of morbidity and mortality may also increase as well as resistance to these effective drugs against malaria.

The recommended actions for improving the quality of malaria treatment may include refresher and fresh training and capacity building for all providers but with special and priority emphasis on patent medicine dealers so as to enhance their quality of drug acquisition. With the recent introduction of ACT in Nigeria, many of the combinations in the markets are not co-formulated but co-packaged and many health providers either prescribe co-packaged drugs or undertake the co-packaging themselves or prescribe two monotherapies with instructions on how to consume them together. Hence, information about the quality of drugs used in the co-packaging or co-prescriptions as ACT is very useful for improving malaria case management. Although advances in forensic chemical analysis and simple field tests will enhance drug quality monitoring, improved access to inexpensive genuine medicines, support of drug regulatory authorities, greater open reporting, vigorous law enforcement, and international cooperation with determined political leadership will be essential to counter this threat [8]. The State Ministry of Health (SMOH) should collaborate with the National Agency for Food, Drug Administration and Control (NAFDAC) to tackle high incidence of counterfeit drugs in the State. It seems that as the war against substandard and counterfeit drugs is being waged in the urban areas by the relevant authorities, the problem continues relatively unabated in the rural areas and amongst low level providers. The Department of Pharmacy in the SMOH in collaboration with the health departments of the local government areas should develop and implement drug quality assurance strategy in the state, which should also lay emphasis on rural areas, in addition to the urban areas.

## Competing interests

The authors declare that they have no competing interests.

## Authors' contributions

OO and HK conceived the study, OO, ND, BU, ES, KH, VO and PO participated in field work. HK carried out the analyses to determine the drug quality. All authors contributed to data analysis. OO wrote the first draft of the paper and all authors participated in the revision until production of the final draft.
